# Health of people with selected citizenships: results of the study GEDA Fokus

**DOI:** 10.25646/11143

**Published:** 2023-03-21

**Authors:** Susanne Bartig, Carmen Koschollek, Marleen Bug, Miriam Blume, Katja Kajikhina, Julia Geerlings, Anne Starker, Ulfert Hapke, Alexander Rommel, Claudia Hövener

**Affiliations:** Robert Koch Institute, Berlin Department of Epidemiology and Health Monitoring

**Keywords:** MIGRATION, HEALTH, SOCIAL DETERMINANTS, DISCRIMINATION, HEALTH INEQUALITY

## Abstract

**Background:**

The health situation of people with a history of migration is influenced by a variety of factors. This article provides an overview of the health of people with selected citizenships using various indicators.

**Methods:**

The analyses are based on the survey ‘German Health Update: Fokus (GEDA Fokus)’, which was conducted from November 2021 to May 2022 among people with Croatian, Italian, Polish, Syrian and Turkish citizenship. The prevalence for each health outcome is presented and differentiated by sociodemographic and migration-related characteristics. Poisson regressions were performed to identify relevant factors influencing health situation.

**Results:**

Self-assessed general health, the presence of depressive symptoms, prevalence of current smoking and the utilisation of general and specialist healthcare differed according to various factors considered here. In addition to sociodemographic determinants, the sense of belonging to society in Germany and self-reported experiences of discrimination were particularly associated with health outcomes.

**Conclusions:**

This article highlights the heterogeneity of the health situation of people with a history of migration and points to the need for further analyses to identify the reasons for health inequalities.

## 1. Introduction

The permanent, cross-border relocation of one’s centre of life – international migration – influences the health of people with their own migration experience and their (direct) descendants born in the country of arrival through various factors before, during and after the biographical event of migration [1–3]. Thus, not only do environmental, sociocultural and (health) political conditions in the country of origin shape the individual’s health, but also the circumstances of migration and experiences related to the migration process itself. For example, people who have fled war zones are more likely to experience traumatic events before and during the migration process than EU citizens who migrate for work-related reasons [4]. On the one hand, the living and working conditions resulting from the socioeconomic status of the country of arrival influence the health of people with (and without) a history of migration (Info box) [2, 5]. On the other hand, there may be specific health risks, such as experiences of discrimination and exclusion, certain – especially legal – access barriers to health services, or psychosocial stresses (e.g., separation from family members) [2, 6]. Consequently, health resources and risks are not solely attributable to migration, but instead require consideration of the circumstances of migration and the associated opportunities for access to societal resources, such as the education system, labour market and healthcare in the country of arrival.

**Info box**

**History of migration, migration background – what terms do we use to describe what?**

People with a migration background or history of migration, immigrants and their (direct) descendants, people with an international history – various terms have been used in recent years to speak about migration and about people living in Germany. In this article, we use the term ‘people with a history of migration’ to refer to people who have immigrated themselves or whose parents have immigrated; however, this term is not intended to replace the statistical category of ‘migration background’.

The concept of ‘migration background’ has been increasingly criticised for multiple reasons, for example, by migrant self-organisations or by the Federal Expert Commission on the Framework Conditions for Integration Capability [22]. Therefore, we suggest that the concept should no longer be applied. On the one hand, the ‘migration background’ is often operationalised in studies in the health sciences differently than in the official statistics. Studies often conflate country of birth and current citizenship [21, 23, 24], whereas the definition of the Federal Statistical Office refers to one’s own and/or parental citizenship at birth [25]. In the general public, the term is often applied without a clear definition and serves to describe people who are German but are supposedly perceived as ‘not from here’. Since its introduction, the term has also experienced a development towards a stigmatising attribution to others [26] and is now mostly rejected as a self-description.

In contrast, the term ‘people with a history of migration’ is often used as a self-description of people who immigrated themselves or whose families have a biographical reference to migration or flight. Again, this term describes a very heterogeneous group of people. Therefore, rather than using aggregate categories such as ‘migration background’ or ‘history of migration’, we recommend analyzing relevant migration-related single indicators combined with other social determinants of health, depending on the particular research question for a differentiated analysis of migration and health [27]. This approach is essential for making differentiated conclusions about factors and explanatory mechanisms of health inequalities.

The description ‘population with a history of migration’ summarizes people who differ both in terms of sociodemographic (age, gender, socioeconomic status) and migration-related characteristics. Thus, health-related opportunities and disease risks vary according to the reasons for migration, the duration of residence as well as the residence status, the language proficiency of the country of arrival, the sense of belonging to society of the arrival country or the subjectively perceived experiences of discrimination. Against this background, it is essential to take into account the heterogeneity within the population of migration history when considering the health situation, including the multiple factors that influence health. However, there are only a few data sources in which people with a history of migration are representatively described and allow differentiated statements on the health situation according to sociodemographic as well as migration-related characteristics [7, 8].

In the project ‘Improving Health Monitoring in Migrant Populations’ (IMIRA I) conducted at the Robert Koch Institute (RKI), measures to improve the integration of people with a history of migration into health monitoring at the RKI were developed and evaluated as part of a feasibility study [9, 10]. The results were implemented in the survey ‘German Health Update: Fokus (GEDA Fokus)’ (data collection: November 2021 – May 2022).

This article aims to describe the health situation of people with Croatian, Italian, Polish, Syrian and Turkish citizenship using selected indicators and taking into account sociodemographic as well as migration-related characteristics based on the GEDA Fokus survey. In addition to indicators of health status (self-assessed general health, presence of depressive symptoms), health behaviour (current smoking) and healthcare (utilisation of general and specialist medical services) are also considered.

Self-assessment of general health (subjective health) is, for example, an important predictor of chronic diseases as well as functional limitations, the utilisation of healthcare services and the risk of mortality [11–15]. The presence of depressive symptoms was selected as an indicator of mental health. Depressive symptoms are not only associated with impaired quality of life and increased morbidity as well as mortality, but also with the use of the healthcare system [16, 17]. Current smoking represents one aspect of health behaviour and is one of the major causes of premature mortality. In addition to damage to the skeletal system, metabolism and periodontium, tobacco use promotes cardiovascular, respiratory and cancer diseases [18–20]. In the utilisation of general and specialist medical services (as indicators of healthcare), specific barriers can affect equal participation in the healthcare system and reinforce health inequalities [2, 6, 21].

Compared to previous research, a key feature of this paper is the differentiated description of respective health outcomes according to a variety of sociodemographic (age, gender, educational status) as well as migration-related characteristics (duration of residence and residence status, sense of belonging to society in Germany, self-reported experiences of discrimination, German language proficiency) to consider the heterogeneity within the population group. In addition to descriptive analyses, influencing factors that are associated with the respective health outcomes are identified using multivariate analyses.

## 2. Methods

### 2.1 Sample design and study implementation

‘German Health Update: Fokus (GEDA Fokus)’ is a multilingual survey of people with selected citizenships (Croatian, Italian, Polish, Syrian and Turkish) that was conducted as part of the project ‘Improving Health Monitoring in Migrant Populations’ (IMIRA II) at the Robert Koch Institute (RKI). The study aimed to collect comprehensive information on health status, health behaviour, living conditions and the utilisation of health services, as well as to enable differentiated statements according to sociodemographic and migration-related characteristics [28]. The (core) indicators developed within the framework of IMIRA I to describe the health situation of people with a migration background formed a thematic focus of the survey content [29]. In addition, relevant migration-sensitive concepts for health monitoring were taken into account, such as subjectively perceived experiences of discrimination or the sense of belonging to society in Germany [30]. Information on COVID-19 infection and vaccination status were also collected [31].

Based on a sample of residents’ registration offices, participants were randomly selected from 99 cities and municipalities throughout Germany according to the characteristic of citizenship (1st, 2nd or 3rd citizenship; accordingly, persons with dual citizenship were included). The selection of five citizenships (population) was based on model calculations using the foreigner statistics [32] and register movements [33] of the Federal Statistical Office from 2015 to 2017. Thus, the size of the citizenship groups, as well as the dynamics of people moving in and out, were considered [28]. The sample population comprised persons between 18 and 79 years of age with Croatian, Italian, Polish, Syrian or Turkish citizenship who had their main residence in one of the selected cities and municipalities at the time of data collection [28]. Persons for whom a conditional blocking notice under § 52 of the Federal Registration Act is deposited in the population register and who are accordingly registered as residing in institutions (e.g., collective accommodation centres for refugees) were included in the sampling.

Data collection was carried out sequentially in a mixed-mode design from November 2021 to May 2022. In addition to a multilingual web-based questionnaire, the participants could participate via a printed paper questionnaire in German or one of the five study languages (Arabic, Croatian, Italian, Polish or Turkish). If there was no response, there was the possibility of a personal interview with partly multilingual interviewers or, in the larger cities, a telephone interview in the preferred language of the participant [28].

A total of 6,038 people (2,983 women and 3,055 men) participated in GEDA Fokus. The response rate was 18.4% (Response Rate 1) according to the standards of the American Association for Public Opinion Research (AAPOR) [34]. The study design of GEDA Fokus is described in detail in another article [28].

### 2.2 Indicators and instruments

This paper presents indicators describing the health status, health behaviour and healthcare utilisation of people with selected citizenships, stratified by sociodemographic as well as migration-related characteristics. The health outcomes were selected based on the (core) indicator system developed within the framework of IMIRA I to describe the health of people with a migration background [29]. Based on various criteria such as public health relevance, informative value as well as national and international dissemination, core indicators for relevant fields of action were identified and consulted with an interdisciplinary panel of experts. In addition to self-assessed general health and the presence of depressive symptoms (field of action: promoting and strengthening health), this article presents the prevalence of current smoking (field of action: promoting and strengthening health-conscious behaviour) and the utilisation of general and specialist medical services (field of action: promoting equal participation in services of the healthcare system). The migration-related characteristics used here follow the recommendations for analysing migration-relevant determinants in public health research in this journal (see Recommendations for collecting and analysing migration-related determinants in public health research).

#### Promoting and strengthening health: history of migration

Subjective health was measured in GEDA Fokus with the question ‘How would you describe your health in general?’ For the analyses, the response options ‘very good’ and ‘good’ were summarised and contrasted with the proportion of participants who rated their health as ‘fair’, ‘bad’ or ‘very bad’.

#### Promoting and strengthening health: depressive symptoms (PHQ-9)

The presence of depressive symptoms was used as an indicator of mental health, measured by the PHQ-9. With this instrument, symptoms of major depression are collected according to the Diagnostic and Statistical Manual of Mental Disorders (DSM-IV, 4th edition) through a questionnaire [35]. The presence of depressive symptoms in the past two weeks is assumed from a sum score of at least ten of the maximum of 27 points.

#### Promoting and strengthening health-conscious behaviour: current smoking

Smoking status was selected to represent one aspect of health behaviour. The question ‘Do you smoke?’ could be answered with ‘yes, daily’, ‘yes, occasionally’, ‘no, not anymore’ and ‘I have never smoked’. In the analyses, a differentiation was made between current smokers (daily or occasionally) and people who do not (no longer) smoke.

#### Promoting equal participation in services of the healthcare system: utilisation of general and specialist medical services

The utilisation of outpatient medical services was surveyed with the question ‘When was the last time you consulted a general practitioner or family doctor on your own behalf?’ (hereafter ‘general medical care’). The question on the utilisation of specialist services used the same wording and the same response options (‘less than 6 months ago’, ‘6 to less than 12 months ago’, ‘12 months ago or longer’, ‘never’). In the analyses, respondents who had used a general practitioner or a specialist in the last 12 months were compared with those who had not sought corresponding medical care during this period.

#### Sociodemographic and migration-related characteristics

The analyses only included people whose gender as reported in the population register matched the gender stated on their birth certificate (according to self-report in the questionnaire). The age of respondents was categorised into the following groups: 18–29 years, 30–44 years, 45–64 years and 65 years and older. Education was categorised into low (ISCED 1–2), medium (ISCED 3–4) and high (ISCED 5–8) groups based on the educational and vocational qualifications of the study participants, according to the 2011 version of the International Standard Classification of Education (ISCED 2011) [36].

As a migration-related characteristic, the duration of residence was categorised into ‘since birth’, ‘up to and including 10 years’, ‘11 to 30 years’ and ‘31 years or more’. The current residence status was operationalised using the following characteristics: ‘German citizenship’, ‘EU citizens’, ‘permanent residence’ and ‘temporary residence’.

The sense of belonging to society in Germany [30] was surveyed with the question ‘How much do you feel you belong to society in Germany?’ The response options were grouped into the following three categories for the analyses: ‘very strongly, strongly’, ‘partly’ and ‘barely, not at all’. In addition, subjectively perceived experiences of discrimination were included in the analyses, which were recorded as follows: ‘How often do any of the following things happen to you in your everyday life?’, ‘You are treated with politeness or less respect than others’, ‘You receive poorer service than other people (e.g., in restaurants or stores)’, ‘Someone behaves as if he or she does not take you seriously’, ‘Someone behaves as if he or she is afraid of you’ and ‘You are threatened or harassed’. Participants who answered ‘very often’, ‘often’ or ‘sometimes’ in one of these areas were grouped together and compared to those who reported ‘rarely’ or ‘never’.

Furthermore, the frequency of self-reported discrimination experiences (‘How often were you treated unfairly or worse than other people in the following situations?’) ‘in the health or care sector (e.g., doctor, hospital, assisted living, care facility)’ was asked [30], which were categorised for the analyses into ‘very often, often’, ‘sometimes’ and ‘rarely, never’.

To map German language proficiency, the responses on mother tongue (‘German’, ‘another language’) and the self-assessed German language skills of those who did not state German as their mother tongue were used and combined into the following categories: ‘mother tongue, very good’, ‘good, moderate’ and ‘poor, very poor’.

### 2.3 Statistical analyses

In the present article, the prevalences of the respective indicators describing the health situation of people with selected citizenships are reported according to sociodemographic and migration-related characteristics with 95% confidence intervals (95% CI). A significant difference between groups (determined by a chi-square test) was assumed when the p-value was less than 0.05. In the following, only the results that were statistically significant according to chi-square tests are reported in the descriptive analyses.

To complement the descriptive analyses, prevalence ratios (PR) and p-values were calculated from Poisson regressions to identify relevant associations between health outcomes and sociodemographic and migration-related characteristics. The regression analyses were adjusted for age, sex and education as well as statistically cross-controlled for the selected migration-related determinants (duration of residence and residence status, sense of belonging to society in Germany, self-reported experiences of discrimination, German language proficiency). Poisson regressions of utilisation of general and specialist medical services included residence status, self-reported experiences of discrimination in the health or care sector, self-assessed German language proficiency and, to take health needs into account, general health. In addition, all Poisson regressions were adjusted for citizenship by registration offices (EMA); however, we refrain from reporting results by individual citizenship groups because, on one hand, the sample composition probably differs systematically between the individual groups, therefore comparability is difficult. On the other hand, the comparison runs the risk of being sweeping and stereotyping when describing individual effects according to citizenship. Complete descriptive and multivariate tables of the results can be found in the Annex.

A weighting factor was included in the analyses to align the sample with the population of corresponding citizenships using the following characteristics: region, gender, age, education (ISCED 2011) and duration of residence. These marginal distributions were taken from the 2018 Microcensus [37] after narrowing the data to the selected five citizenship groups (including dual citizenship). To adequately account for clustering of participants within study locations and weighting when calculating confidence intervals and p-values, survey procedures for complex samples were used in all analyses [38]. The analyses were performed using Stata 17.0.

## 3. Results

###  

#### Sample description

Table 1 shows the sociodemographic and migration-related characteristics of the study population. Of the 6,038 participants, slightly more than half (53.8%) were male (women: 46.2%). The median age among the study population was 42 years. People who belong to the low (45.6%) and medium (40.1%) education groups were represented more frequently within the sample than those of the high education group (14.3%). The largest group of respondents was of Turkish citizenship (26.2%), followed by participants of Polish citizenship (21.5%). Participants with a duration of residence of 31 years or more (29.1%) were represented more frequently in the sample than respondents who have lived in Germany since birth (19.8%). Most of the participants are EU citizens (40.9%), whereas people with temporary residence status were the smallest proportion (13.6%).

Almost two-thirds (63.8%) of the study population reported feeling a very strongly or strongly sense of belonging to society in Germany. Experiences of discrimination were reported by 41.2% of the participants, with the majority (85.8%) rarely or never experiencing discrimination in the health or care sector. While only 7.3% of the study population rated their German language proficiency as very poor or poor, 44.9% assessed themselves at a native-speaker or very good level.

#### Self-assessed health

Overall, 72.3% of participants rated their general health as good or very good, whereby the proportion among women (69.4%) was lower than among men (74.8%) (Annex Table 1). With increasing age, the proportion of those who rated their health as very good or good decreased. While 85.7% of 18 to 29-year-olds assessed their health as very good or good, the proportion among respondents aged 65 years and older was over 40 percentage points lower (42.1%). In addition to gender- and age-specific differences, subjective health varied according to educational status; people in the low education group (63.9%) rated their general health as very good or good less often than those in the medium (77.5%) or high education groups (84.7%). The prevalence of subjective health rated as very good or good, which varied according to educational level (comparison between the low and high education groups), was more pronounced among women (26 percentage points) than among men (16 percentage points).

**Figure 1:**
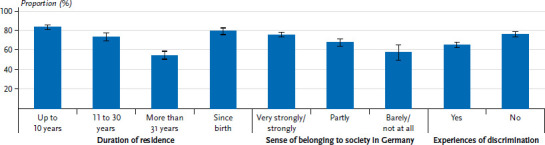
Prevalence of subjective health rated as very good or good by selected characteristics (duration of residence n=5,945, sense of belonging to society in Germany n=5,967, experiences of discrimination n=6,032) Source: GEDA Fokus

General health was assessed as worse with increasing duration of residence in Germany (Figure 1). Respondents who had lived in Germany since birth and those with a duration of residence of up to ten years rated their health as very good or good with similar frequency (80.1% and 84.1%, respectively). In contrast, the proportion of respondents with the longest duration of residence in Germany (31 years or more) was almost 30 percentage points lower (54.9%). Furthermore, there were differences in subjective health according to residence status (Annex Table 1); people with a temporary residence status (77.2%) more often assessed their health as very good or good compared to those with permanent residence status (64.8%). In addition, subjective health varied according to the sense of belonging to German society and subjectively perceived experiences of discrimination (Figure 1). For example, respondents who felt a very strongly or strongly sense of belonging to society in Germany rated their health as significantly better than those who felt barely or not at all a part of it (76.3% versus 57.9%). Participants with self-reported experiences of discrimination were less likely to assess their general health as very good or good compared to people who had never or rarely experienced discrimination (65.8% versus 76.8%).

#### Results of the multivariate analysis: determinants of self-assessed health

To identify relevant factors influencing self-assessed general health, a Poisson regression analysis was conducted. The results largely confirmed the bivariate analyses; female gender, high age (65 years and older) and a low level of education were negatively associated with subjective health rated as very good or good (Annex Table 1). Regarding migration-related characteristics, the duration of residence is a relevant determinant of subjective health. In particular, people with a longer duration of residence (31 years or more) were less likely to rate their health as very good or good compared to those who had lived in Germany for up to ten years (PR=0.78; 95% CI: 0.71–0.85; p<0.001). In addition, respondents who felt that they barely or did not belong to society in Germany (PR=0.73; 95% CI: 0.63–0.84; p<0.001) and participants who reported experiencing discrimination (PR=0.87; 95% CI: 0.82–0.92; p<0.001) assessed their health as worse.

#### Depressive symptoms

Depressive symptoms in the previous two weeks according to PHQ-9 were reported by a total of 20.6% of participants. Women reported depressive symptoms more frequently (23.6%) than men (18.0%) (Annex Table 2). Furthermore, there was an age effect on the prevalence of depressive symptoms. While 29.8% of the youngest age group (18–29 years) reported depressive symptoms, the proportion among respondents aged 65 years and older was significantly lower at 13.4%.

In addition to sociodemographic determinants, the prevalence of depressive symptoms varied according to migration-related characteristics. Thus, participants with a duration of residence of 31 or more years (15.8%) were less affected than those who had lived in Germany since birth (24.0%) or for up to ten years (22.8%). In particular, women who have lived in Germany since birth showed a high prevalence of depressive symptoms compared to male respondents (30.1% versus 18.9%).

**Figure 2:**
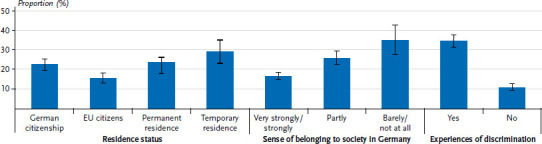
Prevalence of depressive symptoms by selected characteristics (residence status n=5,854, sense of belonging to society in Germany n=5,852, experiences of discrimination n=5,916) Source: GEDA Fokus

Further differences regarding the presence of depressive symptoms can be seen according to residence status (Figure 2). Whereas 15.5% of the EU citizens surveyed reported depressive symptoms, the proportion was almost twice as high among participants with temporary residence status (29.3%). People with German citizenship and participants with permanent residence status were similarly affected (22.6% and 23.7%, respectively). The sense of belonging to society in Germany was also associated with mental health; participants who felt a very strongly or strongly sense of belonging to society in Germany (16.7%) were less likely to report depressive symptoms than those who felt partly (25.9%) or barely or no sense of belonging to society in Germany (35.0%). The prevalence of depressive symptoms also varied according to subjectively perceived experiences of discrimination. Respondents who experienced discrimination were more often affected by depressive symptoms than those who reported never or rarely experiencing discrimination (34.6% versus 10.9%).

#### Results of the multivariate analysis: determinants of depressive symptoms

After adjusting for sociodemographic and migration-related characteristics, gender, age, sense of belonging to society in Germany and subjectively perceived experiences of discrimination were particularly associated with the presence of depressive symptoms (Annex Table 2). Men (PR=0.72; 95% CI: 0.61–0.85; p<0.001) and especially participants aged 65 years and older (PR=0.63; 95% CI: 0.41–0.96; p=0.034) reported depressive symptoms less frequently.

Furthermore, people who felt barely or no sense of belonging to society in Germany showed a 55% (95% CI: 1.20–1.99; p=0.001) higher risk of depressive symptoms than the reference group (very strongly or strongly sense of belonging to society in Germany). Respondents who had experienced discrimination had a 2.77 times higher risk of depressive symptoms than participants who did not experience discrimination (95% CI: 2.30–3.32; p<0.001). In addition, EU citizens reported depressive symptoms less frequently than participants with German citizenship (PR=0.78; 95% CI: 0.63–0.96; p=0.022). Respondents with a duration of residence of 11–30 years were more frequently affected by depressive symptoms than those with a duration of residence of up to ten years (PR=1.36; 95% CI: 1.05– 1.77; p=0.021).

#### Current smoking

Almost one-third (32.9%) of participants stated that they smoke daily or occasionally. Women smoked significantly less often (26.1%) than men (38.7%) (Annex Table 3). Smoking status also varied according to the age and educational status of the respondents. While 18.1% of people aged 65 years and older reported smoking, the proportion was significantly higher among 18 to 29-year-olds (36.4%), especially among male participants at 43.8% (women: 27.1%). In addition, people in the high education group (25.0%) smoked less frequently than those in the low (32.4%) and medium education groups (36.2%). Educational difference (comparison between low and high education groups) in smoking prevalence was more pronounced among men (10.4 percentage points) than among women (4.9 percentage points).

**Figure 3:**
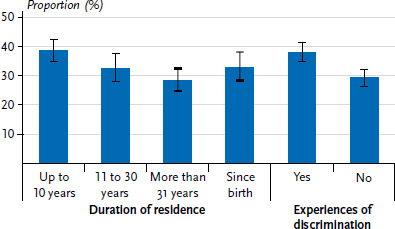
Prevalence of current smoking by selected characteristics (duration of residence n=5,947, experiences of discrimination n=6,034) Source: GEDA Fokus

In addition, the percentage of current smokers differed according to the duration of residence (Figure 3). Whereas 28.5% of participants who had lived in Germany for 31 years or longer smoked at least occasionally, the proportion among respondents with the shortest duration of residence (up to and including 10 years) was almost 10 percentage points higher (38.6%). The prevalence of current smoking, which varied according to the duration of residence, was particularly evident among male respondents. Thus, 47.6% of men who had lived in Germany for up to and including ten years stated that they smoke. In contrast, the proportion of current smokers who have lived in Germany since birth (35.7%) or for 31 years or more (33.4%) was significantly lower. A gender comparison shows that men with a duration of residence of up to and including ten years smoke almost twice as often as women with a similar duration of residence (47.6% versus 25.2%).

Furthermore, participants with German citizenship (27.3%) smoked less frequently than EU citizens (34.3%) and respondents with temporary (36.5%) and permanent residence status (36.0%). Smoking prevalence also varied according to subjectively perceived experiences of discrimination (Figure 3). The proportion of current smokers was almost 10 percentage points higher among participants who reported experiencing discrimination (38.0%) than among those who rarely or never experienced discrimination (29.3%).

#### Results of the multivariate analysis: determinants of current smoking

The multivariate results of the Poisson regression analysis confirm that women, participants aged 65 years and older and people in the high education group were less likely to smoke (daily or occasionally) (Annex Table 3). In addition, self-reported experiences of discrimination were negatively associated with the prevalence of current smoking (PR=1.29; 95% CI: 1.14–1.47; p<0.001). Furthermore, EU citizens smoke more frequently than respondents with German citizenship (PR=1.54; 95% CI: 1.24–1.90; p<0.001).

#### Utilisation of general and specialist medical services

Overall, 77.6% of respondents had used general medical services in the last 12 months, but only half (52.8%) had used specialist medical services. There were gender and age differences in the use of both general practitioner and specialist services, although these were more pronounced for specialist services (Annex Tables 4 and 5). While 62.2% of women stated that they consulted a specialist in the last 12 months prior to the survey, the proportion among men was almost 20 percentage points lower (44.8%). With increasing age, the proportion of respondents who have used general or specialist medical services rises. While among women a continuous increase in specialist services can be observed across the age groups, the use of specialist services among male respondents increased, especially in the middle age groups, from 38.1% among the 30 to 44-year-olds to 56.1% among the 45 to 64-year-olds.

**Figure 4:**
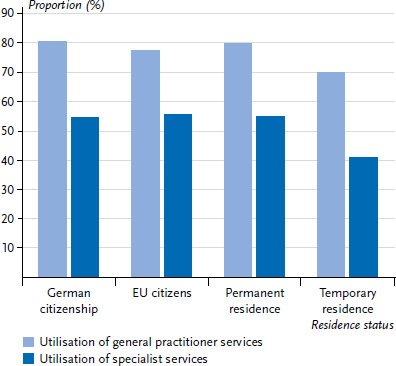
Utilisation of general practitioner (n=5,959) and specialist services (n=5,936) by residence status Source: GEDA Fokus

In addition, utilisation of outpatient medical services varied by self-assessed general health; respondents with (very) good health were significantly less likely to report having used general practitioner (72.5% versus 90.6%) or specialist services (45.2% versus 72.8%) in the 12 months prior to the survey than participants with poorer health. A gender comparison showed that men who rated their health as fair, bad or very bad consulted a specialist less often than women (66.8% versus 78.6%). Regarding the migration-related characteristics, there were differences according to residence status (Figure 4). Among respondents with a temporary residence status, the utilisation of both general (70.0% versus 80.4%) and specialist medical services (40.8% versus 54.6%) was more than ten percentage points lower than that of participants with German citizenship.

#### Results of the multivariate analysis: determinants of the utilisation of general practitioner and specialist services

Compared to women, men showed a lower utilisation of general practitioner (PR=0.90; 95% CI: 0.85–0.94; p<0.001) and especially of specialist services (PR=0.73; 95% CI: 0.68–0.79; p<0.001). Apart from age, self-assessed general health was associated with the utilisation of outpatient medical services (Annex Tables 4 and 5). Participants who rated their health as fair to very bad used general (PR=1.20; 95% CI: 1.15–1.25; p<0.001) and specialist medical services (PR=1.47; 95% CI: 1.34–1.61; p<0.001) more frequently than those with (very) good health. Furthermore, different migration-related characteristics were shown to be determinants of the health services used. Participants who assessed their German language proficiency as very poor or poor (PR=0.89; 95% CI: 0.80–0.98; p=0.016) used general practitioner services less frequently than respondents with a native-speaker or very good language levels (Annex Table 4). Specialist services varied significantly with self-reported experiences of discrimination within the health or care sectors (Annex Table 5). People who rarely or never experienced discrimination in the health or care sectors had a lower use of specialist services than participants who very often or often reported experiences of discrimination (PR=0.79; 95% CI: 0.65–0.94; p=0.011).

## 4. Discussion

This article aims to provide an overview of the health situation of people with Croatian, Italian, Polish, Syrian and Turkish citizenship using selected indicators. Based on analyses of the GEDA Fokus survey, there are clear differences in respective health outcomes according to the sociodemographic and migration-related characteristics considered. The simultaneous control of the effects on the selected indicators also shows that in addition to sociodemographic characteristics, in particular, the sense of belonging to society in Germany as well as subjectively perceived experiences of discrimination are significant determinants of self-assessed general health, depressive symptoms, the prevalence of current smoking and the utilisation of health services. That self-reported experiences of discrimination are associated with both subjective and mental health is confirmed by previous research [39–41]. This suggests that health inequalities are largely attributable to social exclusion mechanisms.

###  

#### Self-assessed health

Almost three-quarters of participants in GEDA Fokus assessed their general health as good or very good, which is a similar proportion to that observed for the German-speaking adult population (69.9%) on the basis of the cross-sectional telephone survey GEDA 2019/2020-EHIS [42]. In addition, comparable gender, age and education differences are found regarding the self-assessed general health for the participants in GEDA Fokus and GEDA 2019/2020-EHIS.

Beyond self-reported experiences of discrimination and a perceived low sense of belonging to society in Germany, a longer duration of residence (31 years or more) was negatively associated with the self-assessed general health. With increasing duration of residence, which naturally goes hand in hand with higher age, the risk of disease increased, which can result from socioeconomic inequality, occupational activities that are hazardous to health (high physical workload) and lower utilisation of health services [2, 43].

#### Depressive symptoms

So far, there are only a few meaningful studies on the mental health of people living in Germany with a history of migration, especially with a refugee experience [44, 45]. Overall, it is pointed out that the mental health of people with migration and refugee experience tends to be worse than that of those without migration history [46–52]. However, it is not the migration process itself that increases the risk for certain mental diseases, but ‘migration-associated stress experiences’ [45]. For instance, people with a history of migration may be exposed to specific psychosocial demands, such as uncertainties regarding their legal residence situation as well as experiences of discrimination and exclusion, which, in combination with less favourable socioeconomic living conditions, can lead to multiple stresses and influence mental health [53–55]. In contrast, people with a history of migration also have psychosocial resources (e.g., social support) that can have a significant influence on stress management and psychological well-being [54, 56].

In accordance with previous studies [46, 48], the present analyses show that women are more likely to be affected by depressive symptoms than men. Contrary to expectations, a temporary residence status is not associated with the presence of depressive symptoms, after reciprocal adjustment of sociodemographic and migration-related characteristics. People who have had refugee experiences are at a higher risk of poorer mental health because migration, which is often forced and not voluntary, can be accompanied by traumatizing experiences before and after the flight. In addition to war-related traumatic experiences, ‘postmigration factors’ can influence mental health [44]. Previous analyses show, for example, that the postmigration stressors of unemployment, loneliness and a rejected or not yet decided asylum application are associated with the presence of depressive symptoms after adjustment for sociodemographic and psychosocial characteristics [44]. Against the background of the higher prevalence of mental diseases among people with a refugee experience, barriers to access to psychiatric, psychotherapeutic and psychosocial care in Germany can lead to health inequalities. In addition to the generally long waiting times for psychotherapeutic services, regardless of the existence of a migration history [57–59], language barriers due to the often unclear reimbursement for interpretation and the restrictive access based on the Asylum Seekers’ Benefits Act (AsylbLG, in particular §§ 4 and 6) can complicate the utilisation of psychotherapeutic services [60–62].

The finding that EU citizens are less likely to have depressive symptoms suggests that migration itself is not a risk factor. A lower prevalence of depressive symptoms among EU citizens compared to the German population has also been found in comparative studies of EU countries [63].

#### Current smoking

A total of 32.9% of the participants in GEDA Fokus stated that they smoke daily or occasionally. Current analyses of the cross-sectional telephone survey GEDA 2019/2020-EHIS indicate a similarly high proportion of smokers in the German-speaking adult population (28.9%) [64]. Regarding smoking prevalences varying by gender, age and education, comparable results emerge as well as women smoke less frequently than men, people aged 65 years and older less frequently than younger people and respondents from the high education group smoke less frequently than people from the low and medium education groups.

The relationship between the duration of residence and smoking status [65, 66], which has been proven in previous research, could not be confirmed based on the present multivariate analyses. Previous studies have shown that the proportion of current smokers decreases with increasing duration of residence, whereby gender-specific differences exist [66–68].

#### Utilisation of general practitioner and specialist services

People with a history of migration can face specific barriers when using services of the health system that make equal participation more difficult. For example, the services offered by the health system are often not oriented towards the diversity and linguistic plurality of the population in Germany [6, 69]. Existing studies indicate that people with a history of migration (especially those with their own migration experience) are less likely to use preventive care such as general health checks, dental check-ups and cancer screening [70–72]. However, there has been insufficient research on the extent to which the different utilisation of health services is due to unequal access or other factors such as different health needs [69].

The present analyses showed that specialist services are used less frequently than general practitioner. In addition to the age effect, gender differences became clear with regard to the health services considered. Women use general and specialist services more frequently than men. Comparable results were reported for the German-speaking adult population based on the cross-sectional telephone survey GEDA 2019/2020-EHIS [73].

The multivariate analyses could not confirm that people with a temporary residence status use general and specialist medical services less often. Based on data from the feasibility study conducted within the framework of IMIRA I, it was shown, among other things, that a temporary residence status and a duration of residence of less than two years are associated with a lower utilisation of general practitioner [74]. Structural barriers resulting from residence status, which complicates access to healthcare, are one of the reasons for this [69]. Accordingly, asylum seekers are confronted with legal restrictions (§ 4 AsylbLG) in their entitlement to healthcare and a more restrictive scope of services [75]. Furthermore, depending on the federal state and partly on the municipalities, differences in the form of grant benefits (treatment voucher versus electronic health card) lead to unequal access to the health system within the group of asylum seekers [76–79] and to health inequalities [80, 81]. Both limited entitlement to health services and ‘health certificate bureaucracy’ [78] represent forms of institutional discrimination [75, 82].

Moreover, the multivariate analyses show that self-assessed German language proficiency are associated with the utilisation of general practitioner services. This is in line with results on the systematic disadvantage of people with a history of migration resulting from linguistically homogeneous care structures of healthcare institutions [69]. For example, lack of multilingual information about health services and language difficulties in communication between health workers and patients can influence both the utilisation and the quality of healthcare [69, 83, 84].

However, there is no effect of self-assessed German language proficiency on the use of specialist care, for which subjectively perceived experiences of discrimination in the health or care sectors are a relevant factor. It should be pointed out, however, that the results do not allow any conclusions to be drawn about causality. For example, whether a higher utilisation of specialist services leads to an increased level of subjectively perceived discrimination or whether a lower utilisation is the consequence of experienced discrimination remains to be determined.

#### Strengths and limitations

The survey study GEDA Fokus offers differentiated analyses according to sociodemographic and migration-related characteristics for a variety of health indicators. In addition to health status, health behaviour or living conditions, detailed information on the utilisation of healthcare and prevention services was collected. Another major strength is the consideration of relevant migration-related concepts, including subjectively perceived experiences of discrimination, sense of belonging to society in Germany or self-assessment of German language proficiency, which were tested within the framework of IMIRA I using cognitive pre-tests and developed accordingly [30]. Consequently, relevant sociodemographic and migration-related factors influencing the health situation of people with selected citizenships can be identified and examined within their heterogeneity and interaction with other social determinants of health.

Nevertheless, it is important to point out an essential limitation that must be considered when describing results. The sampling was based on the characteristic ‘citizenship’, which means that subgroups of the population with a history of migration, such as naturalized persons or people with different citizenships than the five selected, were excluded from the survey. Therefore, extending conclusions from the present sample to the general population of people with a history of migration is not possible based on this selective sample. In addition, the response rate of 18.4% is lower than in the comparable GEDA 2014/2015-EHIS study with 26.9% [85], which was also based on a residents’ registration office sample but targeted the general population. However, the sequential design with the offer of different participation modes in several languages favoured the inclusion of different subgroups so that a possible bias in the willingness to participate could be well countered [86].

#### Conclusion and outlook

This article is an important addition to previous overview publications that described the health situation of people with and without a migration history in a comparative manner. Thus, selected health indicators were presented according to sociodemographic as well as various migration-related characteristics to adequately reflect the heterogeneity of the population group. Against the background of the high importance of such differentiated analyses, the recommendations for the collection and evaluation of migration-relevant as well as social determinants should be given greater consideration in public health research in the future [27]. The differences in individual health outcomes according to the various migration-related and social characteristics shown in this article also point to heterogeneous needs in the conception of measures to improve or strengthen the health situation. Accordingly, diversity-oriented offers of the health system should be intensified.

Based on the available results, subjectively perceived experiences of discrimination are an important determinant of health. Against the background of previous research, there is a lack of empirically-based findings on the extent, forms and effects of individual and institutional discrimination regarding both access to the healthcare system and the quality of healthcare. Additionally, there is a need for systematic research on the extent to which experiences of discrimination and exclusion in the healthcare system influence various health outcomes and thus reinforce health inequalities [69]. In recent years, scientific interest has increased. Research needs regarding racial discrimination and processes of othering have been identified [87, 88] and experiences of discrimination in the healthcare system have been surveyed, for example, within the framework of the Afro Census [89]. The National Discrimination and Racism Monitor (NaDiRa), which is a central project of the German Center for Integration and Migration Research (DeZIM), should also enable reliable statements on the causes, extent and consequences of racism in Germany – including for the area of health – based on various methods [90]. Future research in the field of migration and health should also take greater account of intersectional approaches [27, 69].

## Key statement

- Both the sense of belonging to society in Germany and self-reported experiences of discrimination are associated with subjective and mental health.
- The prevalence of current smoking differs by self-reported experiences of discrimination.
- Specialist medical services are used less frequently than general practitioner services.
- People who rate their German language proficiency as poor or very poor are less likely to use general medical services.
- Experiences of discrimination in the health or care sectors are associated with the utilisation of specialist medical services.

## Figures and Tables

**Table 1:** Sample description by sociodemographic and migration-related characteristics (n=6,038) Source: GEDA Fokus

Number of cases (n)		Weighted sample (%)
**Gender**		
Women	2,983	46.2
Men	3,055	53.8
**Age group**		
18–29 years	1,504	23.5
30–44 years	2,149	33.6
45–64 years	1,818	33.0
**≥**65 years	567	9.9
**Education level**		
Low	1,704	45.6
Medium	2,260	40.1
High	2,042	14.3
Citizenship^*^		
Croatian	1,223	18.0
Italian	1,205	18.9
Polish	1,193	21.5
Syrian	1,209	15.4
Turkish	1,208	26.2
**Duration of residence**		
Up to 10 years	2,474	28.0
11 to 30 years	1,003	23.1
More than 31 years	1,285	29.1
Since birth	1,189	19.8
**Residence status**		
German citizenship	1,563	27.8
EU citizens	2,568	40.9
Permanent residence	818	17.7
Temporary residence	1,025	13.6
**Sense of belonging to society in Germany**		
Very strongly/strongly	3,655	63.8
Partly	1,824	29.1
Barely/not at all	493	7.1
**Experiences of discrimination**		
No	3,572	58.8
Yes	2,466	41.2
**Discrimination experience in the health or care sector**		
Very often/often	225	4.0
Sometimes	608	10.2
Rarely/never	5,167	85.8
**German language proficiency**		
Mother tongue/very good	2,549	44.9
Good/moderate	2,860	47.8
Poor/very poor	538	7.3

^*^according to Residents’ Registration Offices

**Annex Table 1:** Prevalence of subjective health rated as very good or good by sociodemographic and migration-related characteristics (n=5,803) Source: GEDA Fokus

%		(95% Cl)	PR	p-value
Gender^*^				
Women	69.4	(65.9–72.6)	Ref.	
Men	74.8	(72.0–77.4)	1.07	**0.027**
Age group^*^				
18–29 years	85.7	(81.8–88.9)	Ref.	
30–44 years	82.4	(78.9–85.4)	0.92	**0.009**
45–64 years	61.6	(57.2–65.8)	0.74	**<0.001**
≥65 years	42.1	(35.5–48.9)	0.57	**<0.001**
Education level^*^				
Low	63.9	(60.0–67.6)	Ref.	
Medium	77.5	(74.4–80.4)	1.15	**<0.001**
High	84.7	(80.7–87.9)	1.23	**<0.001**
Duration of residence^*^				
Up to 10 years	84.1	(81.1–86.8)	Ref.	
11 to 30 years	74.1	(69.7–78.1)	0.89	**0.004**
More than 31 years	54.9	(50.3–59.4)	0.78	**<0.001**
Since birth	80.1	(75.8–83.7)	0.85	**<0.001**
Residence status^*^				
German citizenship	73.4	(69.4–77.1)	Ref.	
EU citizens	72.6	(68.8–76.1)	1.02	0.594
Permanent residence	64.8	(59.7–69.6)	0.96	0.502
Temporary residence	77.2	(71.9–81.9)	0.91	0.136
Sense of belonging to society in Germany^*^				
Very strongly/strongly	76.3	(73.4–78.9)	Ref.	
Partly	68.2	(64.0–72.1)	0.89	**<0.001**
Barely/not at all	57.9	(49.2–66.2)	0.73	**<0.001**
Experiences of discrimination^*^				
No	76.8	(73.8–79.6)	Ref.	
Yes	65.8	(62.5–69.0)	0.87	**<0.001**

% weighted prevalence, Cl = confidence interval, Ref. = reference group, PR = prevalence ratios

^*^significant differences according to chi-square test

p-values from multivariate Poisson regressions when controlling for each of the selected sociodemographic and migration-related characteristics and adjusted for citizenship by registration offices

Bold = statistically significant in comparison to the reference group

**Annex Table 2:** Prevalence of depressive symptoms in the past two weeks based on PHQ-9 by sociodemographic and migration-related characteristics (n=5,694) Source: GEDA Fokus

%		(95% Cl)	PR	p-value
Gender^*^				
Women	23.6	(21.0–26.5)	Ref.	
Men	18.0	(15.6–20.7)	0.72	**<0.001**
Age group^*^				
18–29 years	29.8	(25.8–34.2)	Ref.	
30–44 years	19.6	(16.9–22.7)	0.78	**0.009**
45–64 years	17.1	(14.1–20.5)	0.72	**0.007**
≥65 years	13.4	(9.1–19.3)	0.63	**0.034**
**Education level**				
Low	21.9	(18.8–25.4)	Ref.	
Medium	19.8	(17.5–22.4)	0.86	0.114
High	18.9	(15.5–22.9)	0.89	0.331
Duration of residence^*^				
Up to 10 years	22.8	(19.2–26.9)	Ref.	
11 to 30 years	21.8	(17.7–26.4)	1.36	**0.021**
More than 31 years	15.8	(13.1–19.1)	1.22	0.219
Since birth	24.0	(20.3–28.2)	1.33	0.073
Residence status^*^				
German citizenship	22.6	(19.6–25.8)	Ref.	
EU citizens	15.5	(13.0–18.4)	0.78	**0.022**
Permanent residence	23.7	(18.8–29.4)	1.30	0.119
Temporary residence	29.3	(23.2–36.3)	1.37	0.096
Sense of belonging to society in Germany^*^				
Very strongly/strongly	16.7	(14.6–18.9)	Ref.	
Partly	25.9	(22.5–29.7)	1.26	**0.015**
Barely/not at all	35.0	(27.5–43.2)	1.55	**0.001**
Experiences of discrimination^*^				
No	10.9	(9.2–12.9)	Ref.	
Yes	34.6	(31.1–38.1)	2.77	**<0.001**

% weighted prevalence, Cl = confidence interval, Ref. = reference group, PR = prevalence ratios

^*^significant differences according to chi-square test

p-values from multivariate Poisson regressions when controlling for each of the selected sociodemographic and migration-related characteristics and adjusted for citizenship by registration offices

Bold = statistically significant in comparison to the reference group

**Annex Table 3:** Prevalence of current smoking by sociodemographic and migration-related characteristics (n=5,804) Source: GEDA Fokus

%		(95% Cl)	PR	p-value
Gender^*^				
Women	26.1	(23.3–29.2)	Ref.	
Men	38.7	(35.4–42.1)	1.46	**<0.001**
Age group^*^				
18–29 years	36.4	(31.9–41.2)	Ref.	
30–44 years	36.3	(32.2–40.5)	1.00	0.981
45–64 years	31.4	(27.7–35.4)	0.89	0.215
≥65 years	18.1	(13.4–24.1)	0.54	**<0.001**
Education level^*^				
Low	32.4	(28.5–36.6)	Ref.	
Medium	36.2	(33.1–39.5)	1.03	0.654
High	25.0	(21.5–28.9)	0.76	**0.005**
Duration of residence^*^				
Up to 10 years	38.6	(34.6–42.7)	Ref.	
11 to 30 years	32.6	(28.0–37.7)	0.90	0.286
More than 31 years	28.5	(24.7–32.7)	0.96	0.693
Since birth	33.0	(28.1–38.3)	1.01	0.920
Residence status^*^				
German citizenship	27.3	(23.7–31.2)	Ref.	
EU citizens	34.3	(31.0–37.6)	1.54	**<0.001**
Permanent residence	36.0	(29.9–42.5)	1.21	0.138
Temporary residence	36.5	(30.0–43.4)	1.04	0.801
**Sense of belonging to society in Germany**				
Very strongly/strongly	32.9	(29.7–36.4)	Ref.	
Partly	33.3	(29.8–36.9)	0.93	0.347
Barely/not at all	30.3	(22.6–39.1)	0.79	0.128
Experiences of discrimination^*^				
No	29.3	(26.3–32.5)	Ref.	Ref
Yes	38.0	(34.7–41.5)	1.29	**<0.001**

% weighted prevalence, Cl = confidence interval, Ref. = reference group, PR = prevalence ratios

^*^significant differences according to chi-square test

p-values from multivariate Poisson regressions when controlling for each of the selected sociodemographic and migration-related characteristics and adjusted for citizenship by registration offices

Bold = statistically significant in comparison to the reference group

**Annex Table 4:** Utilisation of general practitioner in the last twelve months by sociodemographic and migration-related characteristics (n=5,809) Source: GEDA Fokus

%		(95% Cl)	PR	p-value
Gender^*^				
Women	82.8	(79.6–85.6)	Ref.	
Men	73.0	(70.0–75.9)	0.90	**<0.001**
Age group^*^				
18–29 years	73.0	(68.9–76.7)	Ref.	
30–44 years	72.3	(68.5–75.7)	1.00	0.892
45–64 years	83.1	(79.7–86.1)	1.10	**0.001**
≥65 years	87.9	(82.9–91.7)	1.14	**<0.001**
**Education level**				
Low	79.1	(75.1–82.5)	Ref.	
Medium	76.8	(73.7–79.5)	1.00	0.904
High	75.1	(70.9–78.9)	0.99	0.808
Good/very good general health^*^				
Yes	72.5	(69.8–75.1)	Ref.	
No	90.6	(87.8–92.9)	1.20	**<0.001**
Residence status^*^				
German citizenship	80.4	(76.4–83.3)	Ref.	
EU citizens	77.5	(73.9–80.7)	0.95	0.136
Permanent residence	79.6	(74.8–83.8)	1.00	0.925
Temporary residence	70.0	(64.8–74.8)	0.93	0.204
**German language proficiency**				
Mother tongue/very good	79.5	(76.2–82.5)	Ref.	
Good/moderate	76.3	(72.5–79.7)	0.95	0.050
Poor/very poor	73.1	(65.4–79.7)	0.89	**0.016**
**Discrimination experience in the health or care sector**				
Very often/often	83.2	(72.9–90.1)	Ref.	
Sometimes	80.7	(74.4–85.8)	0.97	0.605
Rarely/never	76.9	(74.1–79.4)	0.95	0.359

% weighted prevalence, Cl = confidence interval, Ref. = reference group, PR = prevalence ratios

^*^significant differences according to chi-square test

p-values from multivariate Poisson regressions when controlling for each of the selected sociodemographic and migration-related characteristics and adjusted for citizenship by registration offices

Bold = statistically significant in comparison to the reference group

**Annex Table 5:** Utilisation of specialist services in the last twelve months by sociodemographic and migration-related characteristics (n=5,792) Source: GEDA Fokus

%		(95% Cl)	PR	p-value
Gender^*^				
Women	62.2	(58.7–65.5)	Ref.	
Men	44.8	(41.7–48.0)	0.73	**<0.001**
Age group^*^				
18–29 years	40.4	(36.2–44.8)	Ref.	
30–44 years	48.4	(44.5–52.3)	1.18	**0.004**
45–64 years	61.2	(57.6–64.7)	1.38	**<0.001**
≥65 years	69.8	(63.4–75.4)	1.47	**<0.001**
**Education level**				
Low	53.1	(49.0–57.1)	Ref.	
Medium	52.4	(48.9–55.9)	1.08	0.130
High	53.7	(48.5–58.7)	1.12	0.105
Good/very good general health^*^				
Yes	45.2	(42.2–48.1)	Ref.	
No	72.8	(68.8–76.5)	1.47	**<0.001**
Residence status^*^				
German citizenship	54.6	(49.9–59.1)	Ref.	
EU citizens	55.4	(51.7–59.0)	0.92	0.165
Permanent residence	54.9	(49.2–60.4)	1.09	0.379
Temporary residence	40.8	(35.9–45.9)	0.98	0.884
**German language proficiency**				
Mother tongue/very good	54.0	(50.1–57.9)	Ref.	
Good/moderate	51.5	(48.2–54.8)	0.94	0.182
Poor/very poor	52.0	(44.9–59.0)	0.91	0.183
Discrimination experience in the health or care sector^*^				
Very often/often	67.8	(56.1–77.6)	Ref.	
Sometimes	60.1	(52.5–67.3)	0.86	0.146
Rarely/never	51.4	(48.7–54.0)	0.79	**0.011**

% weighted prevalence, Cl = confidence interval, Ref. = reference group, PR = prevalence ratios

*significant differences according to chi-square test

p-values from multivariate Poisson regressions when controlling for each of the selected sociodemographic and migration-related characteristics and adjusted for citizenship by registration offices

Bold = statistically significant in comparison to the reference group
